# Electrochemiluminescence Aptasensor with Dual Signal Amplification by Silica Nanochannel-Based Confinement Effect on Nanocatalyst and Efficient Emitter Enrichment for Highly Sensitive Detection of C-Reactive Protein

**DOI:** 10.3390/molecules28227664

**Published:** 2023-11-19

**Authors:** Ning Ma, Shuai Xu, Weidong Wu, Jiyang Liu

**Affiliations:** 1 Shanxi Bethune Hospital, Shanxi Academy of Medical Sciences, Tongji Shanxi Hospital, Third Hospital of Shanxi Medical University, Taiyuan 030032, China; mnsxicu@163.com; 2 Tongji Hospital, Tongji Medical College, Huazhong University of Science and Technology, Wuhan 430030, China; 3 School of Chemistry and Chemical Engineering, Zhejiang Sci-Tech University, Hangzhou 310018, China; 202120104178@mails.zstu.edu.cn

**Keywords:** electrochemiluminescence aptasensor, dual dignal amplification, silica nanochannel array film, confinement, C-reactive protein

## Abstract

The rapid and sensitive detection of the important biomarker C-reactive protein (CRP) is of great significance for monitoring inflammation and tissue damage. In this work, an electrochemiluminescence (ECL) aptasensor was fabricated based on dual signal amplification for the sensitive detection of CRP in serum samples. The sensor was constructed by modifying a silica nanochannel array film (SNF) on a cost-effective indium tin oxide (ITO) electrode using the Stöber solution growth method. Gold nanoparticles (AuNPs) were grown in situ within the nanochannels using a simple electrodeposition method as a nanocatalyst to enhance the active electrode area as well as the ECL signal. The negatively charged nanochannels also significantly enriched the positively charged ECL emitters, further amplifying the signal. The recognition aptamer was covalently immobilized on the outer surface of SNF after modification with epoxy groups, constructing the aptasensor. In the presence of CRP, the formation of complexes on the recognitive interface led to a decrease in the diffusion of ECL emitters and co-reactants to the supporting electrode, resulting in a reduction in the ECL signal. Based on this mechanism, ECL detection of CRP was achieved with a linear range of 10 pg/mL to 1 μg/mL and a low limit of detection (7.4 pg/mL). The ECL aptasensor developed in this study offers advantages such as simple fabrication and high sensitivity, making promising applications in biomarker detection.

## 1. Introduction

C-reactive protein (CRP) is an acute phase protein induced by cytokines, primarily synthesized in the liver and regulated by interleukin [1,2]. In healthy individuals, CRP levels are very low (<1 μg/mL), but they rapidly increase during infections, tissue damage, or inflammation. Specifically, serum CRP concentration can rise to more than 500 μg/mL at about 6 h and peak at about 48 h, and the half-life is about 19 h [3]. For instance, sepsis is a severe infectious disease [4]. In patients with sepsis, the levels of CRP are typically elevated. This is because the infection stimulates the immune system to produce an inflammatory response, leading to an increase in CRP synthesis. Therefore, CRP is commonly used as an important indicator, assisting in the diagnosis and treatment monitoring of sepsis. In addition, CRP has been widely used as a non-specific biomarker for inflammation and tissue damage, such as cardiovascular diseases, in clinical medicine [5,6,7]. The American Heart Association (AHA) and the Centers for Disease Control and Prevention (CDC) use serum CRP levels to assess the risk level of cardiovascular diseases. Therefore, the rapid and reliable determination of CRP is of great significance.

Given the value of CRP as a pathological diagnostic indicator in clinical applications, CRP detection has been a research hotspot in the fields of medicine and bioanalytical chemistry. Immunoassay based on the principle of antigen-antibody binding is currently the mainstream technique for CRP clinical monitoring [8,9,10]. However, antibodies themselves have certain limitations, such as complex preparation processes, large batch-to-batch variability, and susceptibility to variability. In recent years, an aptamer-based CRP analysis has attractive attentions. Aptamers are typically short single-stranded oligonucleotide (DNA or RNA) sequences, ranging in length from 25 to 80 nucleotides [11]. In addition to high binding affinity and specificity, aptamers have advantages over traditional antibodies, including ease of modification, good stability, low manufacturing costs, non-toxicity, minimal batch-to-batch variation, reversible folding, and lack of immunogenicity [12]. They also have a wide range of target molecules, including small molecules, peptides, proteins, cells, and tissues. These characteristics make aptamers highly selective bio-recognition elements that have attracted wide attention in constructing various types of biosensors [13,14,15]. During the binding between the recognitive aptamers and target analytes, detection based on aptasensors can be achieved through monitoring optical signals such as fluorescence [16] and surface plasmon resonance (SPR) [17], or electrochemical signals like impedance spectroscopy (EIS), current and voltage changes [18,19], or electrochemiluminescence (ECL) signals [20,21]. Among these, ECL aptaensors offer advantages such as low background noise and high sensitivity. The development of convenient ECL aptasensors for the efficient detection of CRP is highly desirable.

Introducing a catalyst is an effective method to enhance the ECL signal. Commonly used catalysts include metal nanoparticles, enzymes, or organic molecules [22,23,24,25]. These catalysts can undergo specific reactions with an ECL emitter or co-reactor, resulting in stronger luminescent signals. In addition, catalysts can alter the kinetics of ECL reaction, making it more efficient. For instance, gold nanoparticles (AuNPs) possess a large surface area and abundant surface-active sites, promoting electron transfer processes [26] and accelerating the excitation/emission process. It has also been proven that the confinement of metal nanoparticles catalysts within nanospaces provides an effective approach for developing catalysts with high performance [27,28,29]. This is attributed to the fact that confining metal nanoparticles within nanospaces could enhance catalytic activity, selectivity, controllability, and stability. Specifically, precise control over the size and structure of metal nanoparticles catalysts can be achieved by adjusting parameters such as the size, shape, and chemical environment of the nanospaces [30,31,32]. For instance, confining metal nanoparticles within nanospaces provides a smaller size, higher specific surface area and abundant active sites. This facilitates improved contact and reaction rates between the catalyst and reactants, thereby enhancing catalytic activity. At the same time, restricting the diffusion and movement of reactants within nanospaces allows for control over the local environment of the reaction, leading to enhanced selectivity of the reaction. In addition, nanospace confinement can form a protective layer, reducing the interaction between the metal catalyst and the external environment or reactants, and minimizing the deactivation of active sites [33,34,35,36]. This extends the lifetime of the nanocatalyst and enhances its stability in complex reaction systems. Therefore, the utilization of metal nanoparticles confined within nanospaces holds promise for constructing highly sensitive ECL sensing platforms.

The loading and dispersion of metal nanoparticles is also a major difficulty. Porous materials have extensive applications in fields such as separation, catalysis, energy, and sensing due to their high specific surface area, permeability, tunability, and strength stability [37,38,39]. Silica nanochannel array film (SNF) is a thin nanofilm with nanoscale channel-like structures, offering tunability, high selectivity, and stability [40,41,42,43]. On the one hand, the pore size and film thickness of SNF can be adjusted by controlling the preparation conditions. The uniformly distributed nanochannels enable selective filtration, separation, or transport of molecules or ions of specific sizes or shapes [44,45,46,47]. This high selectivity gives them great potential in selective enrichment, separation, and filtration applications. For instance, the ionization of silicon hydroxyl groups (p*K*_a_~2–3) on the SNF surface results in a negatively charged surface that can significantly enrich positively charged ions [48,49,50,51]. Furthermore, the high specific surface area of the densely packed ultrasmall nanochannels enhances this enrichment effect [52]. On the other hand, SNF exhibits excellent chemical and thermal stability due to their robust silica structure. Thus, SNF exhibits great potential in constructing high-performance ECL sensing platforms.

In this study, an electrochemiluminescence (ECL) aptasensor platform was constructed by combining the confinement effect of silica nanochannel array film (SNF) on nanocatalysts and the efficient enrichment of ECL emitters, which can be applied for the highly sensitive determination of CRP. Using a cost-effective and readily available ITO electrode as the supporting electrode, SNF was grown on ITO surface through the Stöber solution growth method. Confined gold nanoparticles (AuNPs) were in situ electrodeposited within the nanochannels as nanocatalysts. After the outer surface of SNF were derivatized by the epoxide group, the recognitive interface could be obtained after covalent immobilization of amino-modified aptamers followed by blocking the non-specific sites. The ECL signal could be enhanced combined with the electrostatic attraction of NH_2_-SNF towards the ECL emitter, Ru(bpy)_3_^2+^, and the increased electrode active area as well as a catalytical process by AuNPs. Because the formed aptamer-CRP complex hinders the diffusion of the ECL emitter and co-reactant into the nanochannel array as well as the underlying electrode, it leads to a decrease in the ECL signal. Based on this principle, the ECL determination of CRP can be achieved. The constructed aptasensor also exhibits the advantages of a simple preparation method, high sensitivity, stability, and selectivity.

## 2. Results and Discussion

### 2.1. Construction of Aptasensor and CRP Detection Based on Dual Signal Amplification

**Figure 1:**
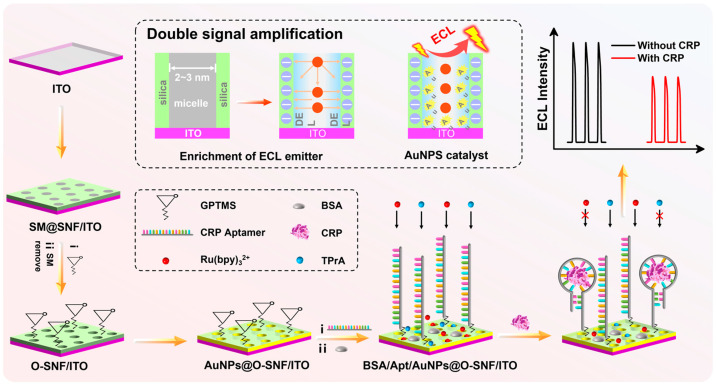
Illustration of the fabrication of the aptasensor and ECL detection of CRP based on dual signal amplification.

The construction of the aptamer-based sensor and ECL determination of CRP based on dual signal amplification are shown in Figure 1. Firstly, a layer of SNF is grown on the patterned ITO electrode surface using the Stöber solution growth method. The growth mechanism involves the self-assembly of siloxane precursors under the surfactant micelle (SM) template, resulting in SM within the nanochannels of the obtained electrode (SM@SNF/ITO). Subsequently, the electrode is epoxy-functionalized. Since the nanochannels are sealed by SM, the reaction occurs selectively on the outer surface of SNF, leading to the electrode being abbreviated as SM@O-SNF/ITO. The sealed SM prevents an epoxy reaction inside the nanochannels, avoiding a blockage of the nanochannels. After removing SM, open nanochannels are obtained (O-SNF/ITO).

By placing the O-SNF/ITO electrode in a chloroauric acid solution for in situ electro-deposition, gold nanoparticles are deposited inside the nanochannels [34], resulting in the AuNPs@O-SNF/ITO electrode. Then, amino-functionalized aptamers undergo a covalent binding with the epoxy groups on the external surface of the nanochannels, forming the recognition and capture interface for CRP. After blocking the non-specific active site with BSA, the aptasensor, BSA/Apt/AuNPs@O-SNF/ITO, is constructed. The main role of BSA is to prevent non-specific binding through blocking the remaining space on the electrode surface after fixing the recognitive ligands. When the target substance, CRP, is present, it binds to the aptamer on the electrode surface. The formed large-sized complex obstructs the diffusion of the ECL probe Ru(bpy)_3_^2+^ and the co-reactant tri-n-propylamine (TPrA) through the nanochannels to reach the underlying electrode, resulting in a decrease in ECL signal. Based on this mechanism, ECL detection of CRP can be achieved.

Figure 1 also illustrates the dual signal amplification during ECL detection of CRP. On one hand, SNF features a vertically oriented array of silica nanochannels on the supporting electrode. The inner wall of nanochannel contains a large number of silanol groups with low p*K*_a_ (2~3), which ionize in common buffer systems, forming negatively charged sites that significantly enrich the positively charged ECL probe Ru(bpy)_3_^2+^ through electrostatic attraction, leading to a significantly enhanced ECL signal [53]. On the other hand, AuNPs deposited inside the nanochannels serve as nanocatalysts to enhance the signal of the Ru(bpy)_3_^2+^/TPrA system [28]. When CRP binds to the aptamer to form a complex on the outer surface of SNF (e.g., the entrance of nanochannel), the steric hindrance effect prevents the ECL emitter Ru(bpy)_3_^2+^ and the co-reactive agent TPrA from reaching the electrode surface, affecting the mass transfer and electron transfer of the probe. Thus, the ECL signal decreases. Based on this mechanism, CRP can be detected.

### 2.2. Characterization of the SNF/ITO and AuNPs@SNF/ITO Electrodes

**Figure 2:**
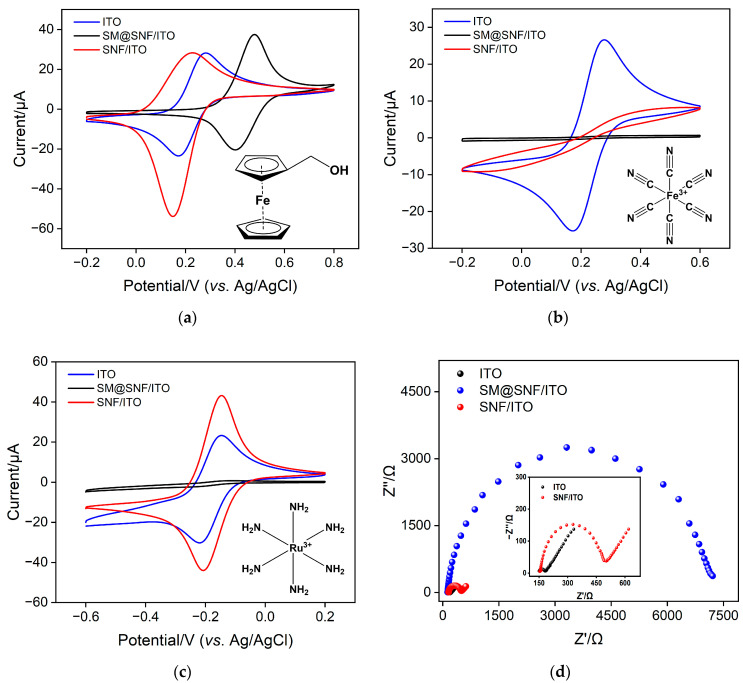
CV curves obtained on ITO, SM@SNF/ITO, and SNF/ITO in 50 mM KHP containing 0.5 mM (**a**) Fc-MeOH, (**b**) Fe(CN)_6_^3−^ and (**c**) Ru(NH_3_)_6_^3+^. (**d**) EIS plots of SM@SNF/ITO and SNF/ITO in a 0.1 M KCl solution containing 2.5 mM Fe(CN)_6_^3−/4−^. The scan rate in (a), (b) and (c) was 0.05 V/s.

The electrochemical properties of the SNF film grown on the electrode surface were characterized. The electrochemical signals of three redox probes with various charges including (neutral, Fc-MeOH; negatively charged Fe(CN)_6_^3−^, positively charged Ru(NH_3_)_6_^3+^) were used to investigate the integrity and charge-selective permeability of the SNF film (Figure 2a–c). When SM@O-SNF/ITO was placed in a FcMeOH probe solution, SM@O-SNF/ITO exhibited an extraction effect on FcMeOH due to the hydrophobic interactions between FcMeOH and SM [40]. As shown in Figure 2a, the SM@O-SNF/ITO electrode showed a pair of oxidation-reduction peaks, with the peak potential shifting towards the positive direction compared to that obtained on an ITO electrode. Since the oxidation product of FcMeOH carries a positive charge and can be enriched by the negative-charged nanochannels, the reduction peak current of FcMeOH on O-SNF/ITO is greater than the oxidation peak current [40]. Due to the presence of hydrophobic SM inside the nanochannels, SM@O-SNF/ITO shows no signal in the solutions of Fe(CN)_6_^3−^ (Figure 2b) and Ru(NH_3_)_6_^3+^ (Figure 2c) probes, only exhibiting a charging current. This indicates the complete coverage of SNF on the ITO surface with SM-blocked nanochannels. After the removal of SM, the electrode displays significant Faradaic current in the charged redox probes. Compared to ITO, SNF/ITO exhibits apparent suppression of Faradaic current in the anionic Fe(CN)_6_^3−^ solution. On the contrary, an enhancement current is observed in the case of cationic Ru(NH_3_)_6_^3+^ probes. This is because the SNF surface and nanochannels contain numerous silanol groups, causing a negative charge in the detection medium. This negative charge can repel anions while attracting cations through electrostatic interactions, demonstrating significant charge-selective permeability. The electrochemical impedance spectroscopy (EIS) plots of the different electrode are shown in Figure 2d. As shown, SM@SNF/ITO electrode displays a significantly high electron transfer resistance (*R*et) of 7057 Ω, which results from hindered electron transfer between the probe and the electrode due to the sealing of nanochannels by SM. When SM was removed, *R*et obtained on SNF/ITO remarkably decreased (336 Ω) because of the open nanochannels, which is also higher than that of ITO (29 Ω) owing to the silica structure. These results also prove the successful modification of SNF on the ITO electrode.

**Figure 3:**
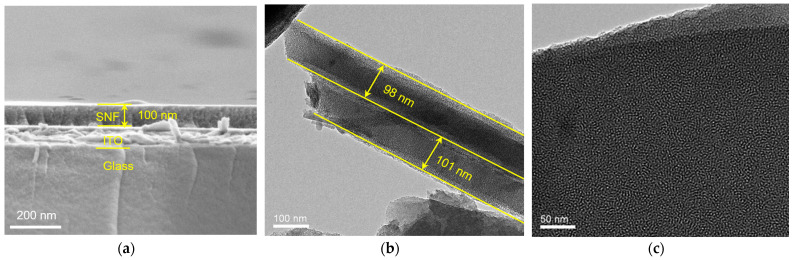
(**a**) SEM image of the cross-section of SNF/ITO. The cross-sectional (**b**) and top-view (**c**) TEM images of SNF.

The morphology of SNF-modified ITO electrodes was characterized using scanning electron microscopy (SEM). As commonly known, the ITO electrode, also known as ITO conductive glass, is formed by depositing an ITO layer on the surface of glass. In this work, ITO electrodes were used as the support electrode and further modified with SNF. Figure 3a gives the SEM image of the cross-section of the SNF/ITO electrode. As shown, ITO/SNF displays a three-layer structure including the glass substrate, the ITO layer, and the SNF layer from bottom to top. Since ITO electrodes cannot be characterized by transmission electron microscopy (TEM), SNF was carefully scraped off using a scalpel, followed by ultrasonication, and then drop-cast onto a copper grid for TEM characterization. Figure 3b depicts the cross-sectional TEM images of two separate layers of SNF. Each SNF is composed of mutually parallel nanochannels with a thickness of approximately 100 nm. Figure 3c displays the top-view TEM image of SNF. As observed, SNF has densely packed nanochannels, as each bright spot represents a nanochannel. The average diameter of the nanochannels is 2.7 nm. Within the observed range, SNF shows no cracks.

**Figure 4:**
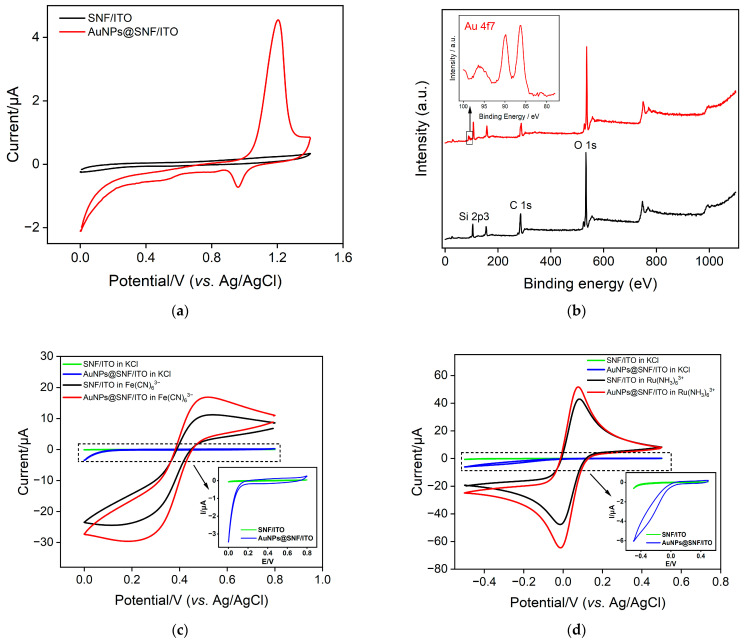
(**a**) CV curves of SNF/ITO and AuNPs@SNF/ITO in 0.1 M H_2_SO_4_ solution. (**b**) XPS profiles of SNF/ITO (black) and AuNPs@SNF/ITO (red). (**c**) CV curves of SNF/ITO and AuNPs@SNF/ITO in KCl solutions in absence or presence of 1 mM (**c**) Fe(CN)_6_^3−^ and (**d**) Ru(NH_3_)_6_^3+^. Inset is the amplified figure of curves without probe.

To confirm the successful deposition and localization of AuNPs, the SNF/ITO electrode before and after AuNPs’ deposition (AuNPs@SNF/ITO) was investigated using cyclic voltammetry (CV) and X-ray photoelectron spectroscopy (XPS). The CV curves of both electrodes are shown in Figure 4a. The SNF/ITO electrode exhibits virtually no signal in 0.1 M of sulfuric acid, while AuNPs@SNF/ITO shows characteristic oxidation-reduction peaks of gold at 1.18 V and 0.96 V, respectively. In comparison with SNF/ITO, characteristic peaks of Au_4f_ appear in the XPS spectrum for AuNPs@SNF/ITO, further confirming the successful modification of AuNPs (Figure 4b). Figure 4c,d show the CV curves of SNF/ITO and AuNPs@SNF/ITO electrodes in the absence or presence of different probe solutions. It can be found that the charging current and peak current of the AuNPs@SNF/ITO electrode are higher than those of the SNF/ITO electrode for both positive and negative probes. This indicates that the accessible electrode area is improved after the modification of AuNPs.

**Figure 5:**
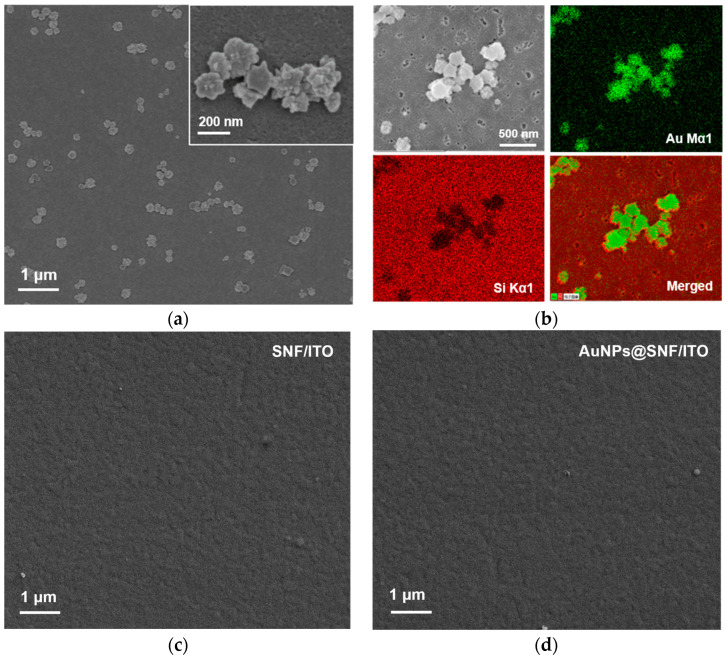
(**a**) SEM image of the electrode surface after removal of SNF. Inset is the corresponding high-resolution SEM image. (**b**) Elemental mapping of the surface of AuNPs@SNF/ITO after dissolving SNF. The image in the top left is the SEM photograph of the region where element mapping was conducted. The top right image is the gold element mapping, the bottom left image is the silicon element mapping. Green and red correspond to signal points of Au or Si elements, respectively. The bottom right image is an overlay of gold and silicon mapping signals. (**c**) SEM image of SNF/ITO. (**d**) SEM overhead image of AuNPs@SNF/ITO.

Due to the ultrasmall nanochannels of SNF, the size of in situ grown AuNPs is relatively small. The surface morphology of the obtained electrode was determined by dissolving the SNF membrane. Figure 5a,b show SEM and elemental mapping images of the electrode surface after removing SNF using sodium hydroxide solution. From the images, it can be observed that the nanoparticles appear irregular and spiky, and they tend to aggregate (Figure 5a). The elemental mapping images confirm the Au element (Figure 5b). As SNF is dissolved, the small AuNPs undergo aggregation, leading to the formation of larger-sized gold materials. SEM images of SNF/ITO before and after AuNPs deposition have been investigated. SNF/ITO surfaces remain smooth and flat before (Figure 5c) and after (Figure 5d) modification of AuNPs. Thus, AuNPs exposed outside the nanochannels are not observed. After combining the signal peak for the gold element in XPS characterization, which has a testing depth of only a few to several tens of nanometers, and the gold element mapping result after dissolving the SNF film, it is speculated that the deposited AuNPs are on the electrode surface or within the nanochannels.

### 2.3. Dual Signal Amplification Based on Enrichment of ECL Emitter and AuNPs’ Nanocatalyst

**Figure 6:**
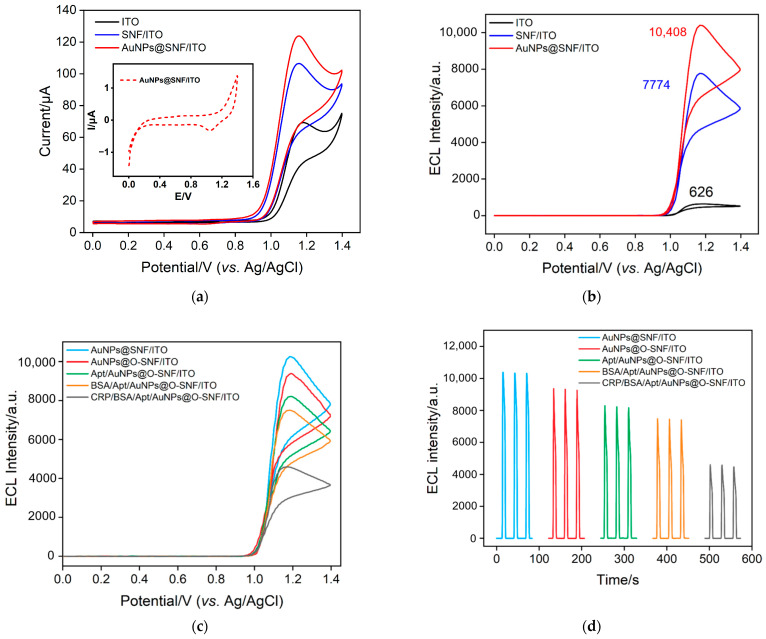
(**a**) CV curves obtained on ITO, SNF/ITO and AuNPs@SNF/ITO electrodes in 0.01 M PBS (pH 7.4) solution containing 10 μM Ru(bpy)_3_^2+^ and 3 mM TPrA. Inset is the CV of AuNPs@SNF/ITO in blank PBS. (**b**) ECL signal plots of ITO SNF/ITO and AuNPs@SNF/ITO electrodes in 0.01 M PBS (pH 7.4) solution containing 10 μM Ru(bpy)_3_^2+^ and 3 mM TPrA. ECL intensity-potential curves (**c**) and ECL intensity-time curves (**d**) were obtained at different electrodes. Scan rate of CV: 100 mV s^−1^. Scan potential: 0–1.4 V. Photomultiplier tube (PMT) voltage was 400 V.

Figure 6a presents the CV curves obtained on ITO, SNF/ITO, and AuNPs@SNF/ITO in a Ru(bpy)_3_^2+^/TPrA solution. No oxidation peak for gold around 1.2 V was observed. Additionally, the CV curve of AuNPs@SNF/ITO in blank PBS has also been added as an inset of Figure 6a. No gold oxidation peak at ~1.2 V was observed and only a weak reduction peak with very low current near 1.05 V was observed. These results confirm that the deposited gold remains stable during the ECL measurement and does not produce interfering electrochemical signals. The corresponding ECL signals measured on the three types of electrodes are shown in Figure 6b. Compared to bare ITO, the CV oxidation peak of the SNF-modified electrode significantly increases. It can also be observed that SNF/ITO exhibits a 12.4-fold enhancement in ECL intensity compared to the ITO electrode, resulting from the enrichment of negatively charged nanochannels on positively charged Ru(bpy)_3_^2+^. Thus, the significant enrichment of the ECL emitter by nanochannels can markedly improve the ECL signal. When AuNPs were further in situ modified, the oxidation-reduction peak current in the CV curve further increased, and the ECL intensity increased by an additional 34% compared to SNF/ITO, demonstrating that AuNPs can act as nanocatalysts to enhance the ECL signal. This may be attributed to the electrocatalytic oxidation of TPrA by AuNPs, generating more TPrA cationic-free radicals (TPrA^+^), promoting the generation of more excited states and enhancing the ECL signal.

### 2.4. Feasibility of the Construction of the Aptasensor

To investigate the feasibility of fabricating the aptasensor, the electrodes obtained in the stepwise modifications were investigated through electrochemical methods. As shown in Figure 6c,d, the signal slightly decreased. Upon covalently attaching the aptamer to the surface of the AuNPs@O-SNF/ITO electrode, the ECL signal of the Apt/AuNPs@O-SNF/ITO electrode decreased. This can be attributed to the potential hindrance of the aptamer attachment, which may obstruct the entry of co-reactants and ECL probes into the nanochannels, resulting in a reduction in the ECL signal.

After blocking non-specific sites with BSA, the electrode surface was covered with a non-conductive, large-sized protein layer, further impeding the transfer of probes and electron transfer, resulting in a further decrease in the ECL signal of BSA/Apt/AuNPs@O-SNF/ITO. In the presence of CRP, the formation of complexes due to CRP binding to the aptamer further hindered probe transfer and electron transfer, causing a significant reduction in the ECL signal. These results confirm the successful construction of the aptasensor and the feasibility of CRP detection.

### 2.5. Optimization of Experimental Conditions

**Figure 7:**
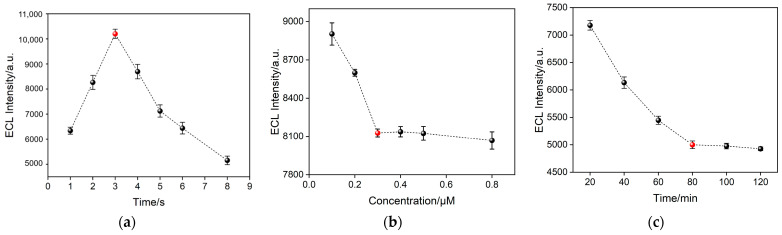
(**a**) Effect of deposition time of AuNPs on ECL signal of AuNPs@O-SNF/ITO electrode. (**b**) Optimization of the aptamer concentration on the ECL signal. (**c**) Optimization of CRP (1 ng/mL) incubation time on the ECL signal. Electrolyte solution: 0.01 M PBS (pH 7.4) solution containing 10 μM Ru(bpy)_3_^2+^ and 3 mM TPrA. Scan rate: 100 mV s^−1^. Scanning potential: 0~1.4 V. PMT = 400 V. The red dots represent the optimized conditions.

To achieve high detection performance of the fabricated aptasensor, certain relevant conditions for the experiment were optimized. The deposition time of AuNPs, the concentration of the aptamer for the fabrication of the recognitive interface, the incubation time for CRP, and the results are shown in Figure 7. As observed in Figure 7a, compared to the electrode without AuNPs (deposition time of 0 s), the ECL signals of the electrode exhibit an initial increase followed by a decrease as the AuNPs deposition time increases. The increase in ECL signal is attributed to the enhanced electrode active surface area due to the presence of AuNPs, which also catalyze the ECL luminescence process. As a prolonged deposition time may lead to blockage within the nanochannels, resulting in reduced enrichment of Ru(bpy)_3_^2+^ by the nanochannels and subsequently a decrease in the ECL signal of the sensor, the optimal deposition time for AuNPs was chosen at 3 s (Figure 7a). The modification of the bio-recognitive interface is crucial for the fabrication of the aptasensor, taking into account both time and cost factors. When the incubation time for aptamer immobilization was fixed at 90 min [54], the aptamer concentration was optimized. As shown in Figure 7b, the fixed incubation time was 30 min, and ECL signal decreases with increasing aptamer concentration and stabilizes at around 0.3 μM. Further increasing the aptamer concentration only results in minimal changes in the ECL signal. Thus, the aptamer concentration of 0.3 μM was chosen as the optimal condition. Similarly, in the case of antigen concentration of 1 ng/mL, the optimal incubation time for CRP, as obtained through optimization, was 80 min (Figure 7c).

### 2.6. ECL Determination of CRP

**Figure 8:**
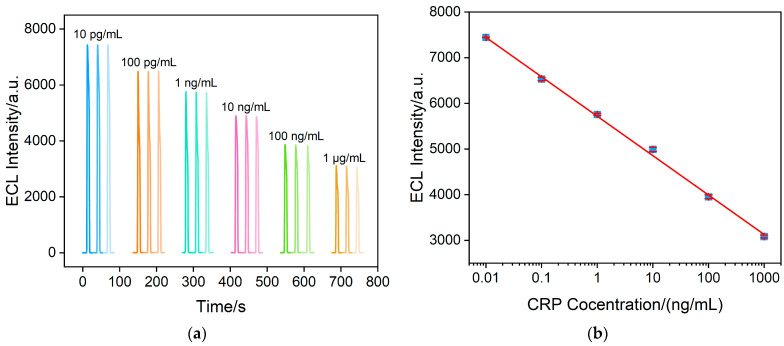
(**a**) ECL intensity of the aptasensor at different CRP concentrations. (**b**) Calibration curves of ECL intensity versus CRP concentration in the range from 0.01 to 1000 ng/mL. The error bars show the standard deviation of measurements taken from three experiments.

Under optimal conditions, the proposed aptasensor exhibits a negative correlation between ECL intensity and CRP concentration (Figure 8a). Commonly, logarithmic processing is usually performed on the electrochemical data to explore a good coefficient of determination across a wide concentration range [55]. As observed from Figure 8b, within the range from 10 pg/mL to 1 μg/mL, the ECL signal (*I*_ECL_) demonstrates a linear relationship with the logarithm of CRP concentration (log*C*_CRP_). The corresponding linear regression equation (*I*_ECL_ = −863.3 log*C*_CRP_ + 5717) exhibits a high linear correlation coefficient of 0.9995. The detection limit (DL) calculated using three signals to noise (S/N = 3) is 7.4 pg/mL. Comparison of CRP detection performance with other sensors is shown in Table S1 (SI) [18,56,57,58,59,60,61]. The LOD of the fabricated aptasensor is lower than that of electrochemical sensors using square wave voltammetry [56] or differential pulse voltammetry [57], or ECL immunosensors based on platinum nanowire/titania nanotube composites [58] or Ir(III) compound and β-cyclodextrin complex [59]. The LOD is higher than that of the ECL immunosensor based on the resonance energy transfer of Ru(bpy)_3_^2+^@Cu_3_(HHTP)_2_ and graphene oxide-gold nanoparticles (GO-AuNPs) [60], an ECL immunosensor using Ru(bpy)_3_^2+^-labeled AuNPs’ modified screen-printed electrode (SPE) combined with a flow measurement [61], or an electrochemical aptasensor based on AuNPs anchored in covalent poly deep eutectic solvent functionalized graphene-modified glassy carbon electrode (Apt/AuNPs/GO/PDES/GCE) [18]. However, the fabricated aptasensor does not conducted a complex preparation of nanocomposite materials or labelled bioligands and exhibits a wider detection range.

### 2.7. Specificity, Selectivity, Repeatability and Stability of the Fabricated Aptasensors

**Figure 9:**
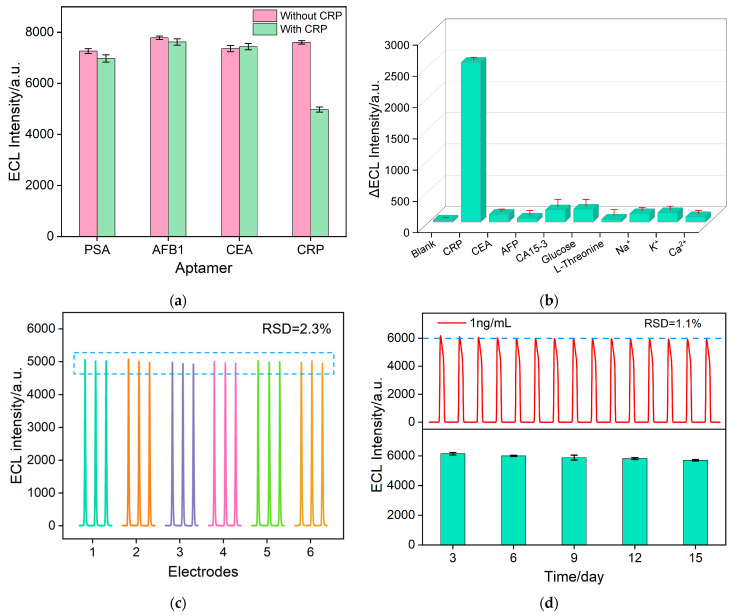
(**a**) ECL signal measured before and after incubation with 10 ng/mL CRP in PBS solution containing 10 μM Ru(bpy)_3_^2+^ and 3 mM TPrA. The recognitive interface was fabricated using different aptamer PSA, AFB1, and CEA. (**b**) ECL signal change values of BSA/Apt/AuNPs@O-SNF/ITO after incubation with 10 ng/mL CRP or other substances. The concentrations were 100 ng/mL for CEA, 100 ng/mL for AFP, 100 ng/mL for CA15-3, 100 μM for Glucose, 100 μM for L-Threonine, 1 mM for Na^+^, 1 mM for k^+^ and 1 mM for Ca^2+^. (**c**) The reproducibility of the aptasensor. ECL intensity after incubation with different batches of six electrodes and 10 ng/mL CRP. (**d**) ECL signal stability of the aptasensor for a single measurement.

Other aptamers with an amino label, such as the AFB_1_ aptamer, PSA aptamer, and CEA aptamer, were employed to assess the specificity of the sensor. As shown in Figure 9a, only sensors modified with the CRP aptamer exhibit significant signal changes. Selectivity is also a crucial parameter for assessing sensor performance. Figure 9b displayed the change of ECL signal (ΔECL intensity) when the aptasensor incubated with CRP (10 ng/mL), or one (100 ng/mL) of the other tumor biomarkers (AFP or CA15-3) or substances (glucose, L-serine, Na^+^, K^+^ and Ca^2+^) found in serum. As observed, even the concentrations of other substances are ten times higher than that of CRP, and the change of ECL signal is very minimal. In contrast, a significant ECL change is observed when the aptasensor incubates with CRP because of the decrease in ECL signal resulting from the specific binding with aptamer, indicating that the aptasensor possesses excellent selectivity. Six aptasensors are individually fabricated to measure CRP, as shown in Figure 9c. The results indicated a relative standard deviation (RSD) of 2.3%, demonstrating excellent repeatability of the sensors (Figure 9c). As shown in the upper image in Figure 9b, when the electrode undergoes continuous electrochemical scanning, the RSD of the continuously measured ECL signals is only 1.1%, demonstrating high signal stability. Furthermore, even after 15 days of storage at 4 °C, the ECL intensity of the sensors remains at 85% of the initial signal, indicating excellent storage stability (the bottom image of Figure 9d).

### 2.8. Real Sample Analysis

Compared to turn-on sensors, turn-off sensors are more susceptible to matrix effects when analyzing samples with complex matrices, thereby affecting detection sensitivity and accuracy. The potential application of the aptasensor in measuring CRP in real biological samples was assessed through standard addition experiments conducted in serum. The easy detection of CRP levels even when the serum is diluted to 1/50 is achieved. After incubating the sensor with serum containing 0.1–100 ng/mL CRP, ECL signals were measured, and recovery rates were calculated. The results are shown in Table 1. As shown, the recovery ranges between 94.4% and 104%, with a low RSD of less than 2%, indicating high detection reliability. Thus, the impact of complex matrices on the detection is low thanks to the size exclusion and charge-selective permeability of the employed nanochannel array. Although the dilution of the sample diminishes the convenience of the detection process compared with turn-on sensors, the proposed strategy can potentially be extended to the detection of other biomolecules with a simple replacement of the corresponding aptamer.

## 3. Materials and Methods

### 3.1. Chemicals and Materials

The recombinant human CRP protein was purchased from Okay Biotechnology Co., Ltd. (Nanjing, China). Carcinoembryonic antigen (CEA), bovine serum albumin (BSA), alpha-fetoprotein (AFP), and cancer antigen 15-3 (CA15-3) were obtained from Key-Bio Biotech Co., Ltd. (Beijing, China). Human blood serum from a healthy male donor was provided by the Hangzhou Institute of Occupational Diseases (Hangzhou, China). The CRP aptamer, PSA aptamer, CEA aptamer, and AFB_1_ aptamer with amino labeling at the 5′ end were purchased from Sangon Biotech Co., Ltd. (Shanghai, China). The aptamer sequence is shown below: CRP (5′-NH_2_-CGA AGG GGA TTC GAG GGG TGA TTG CGT GCT CCA TTT GGT G-3′); PSA (5′-NH_2_-ATT AAA GCT CGC CAT CAA ATA GCT GC-3′); CEA (5′-ATACCAGCTTATTCAATT-NH_2_-3′); AFB1 (5′-GTT GGG CAC GTG TTG TCT CTC TGT GTC TCG TGC CCT TCG CTA GGC CCA CA-NH_2_-3′). Chemicals, including potassium ferrocyanide ([K_4_Fe(CN)_6_], 99%), ethanol (EtOH, ≥99.7%), tri-n-propylamine (TPrA, 98.0%), hydrochloric acid, hydroxymethyl ferrocene (FcMeOH, 98%) and potassium ferricyanide ([K_3_Fe(CN)_6_], 98%), were purchased from Aladdin Reagents (Shanghai, China). Hexaammineruthenium (III) chloride ([Ru(NH_3_)_6_]Cl_3_, 98%), (3-glycidyloxypropyl) trimethoxysilane (GPTMS), tetraethyl orthosilicate (TEOS, ≥99%) and hexadecyl trimethylammonium bromide (CTAB, 99%) were sourced from Sigma–Aldrich (Shanghai, China). Tripropylamine and potassium hydrogen phthalate (KHP, 99%) were obtained from Maclin Biochemical Technology Co., Ltd. (Shanghai, China). Indium tin oxide (ITO)-coated glasses were purchased from Kaivo Optoelectronics Technology Co., LTD. (Zhuhai, China). The dilution of biological reagents and the detection of CRP were carried out in phosphate buffer solution (PBS, 0.01 M, pH = 7.4) obtained with KH_2_PO_4_ and Na_2_HPO_4_. Ultrapure water (18.2 MΩ·cm) was prepared using a Milli-Q water purification system. All chemicals were used as received without further purification.

### 3.2. Characteriaztions and Instrumentations

The film’s microscopic structure and morphology were examined using a transmission electron microscope (TEM, HT7700, Hitachi, Japan) and field-emission scanning electron microscope (SEM, SU-8010, Hitachi, Japan), respectively. AuNPs were characterized using X-ray photoelectron spectroscopy (XPS) with a PHI5300 instrument, employing a Mg Kα source excitation at 250 W and 14 kV. Elemental mapping was conducted with an Oxford Xplore 50 energy spectrometer. The acceleration voltage was 15 kV. Cyclic voltammetry (CV) measurements were conducted on an Autolab electrochemical workstation (PGSTAT302N, Metrohm, Switzerland). A three-electrode system was employed, consisting of Ag/AgCl as the reference electrode, Pt electrode as the counter electrode, and ITO or modified ITO as the working electrode. ECL measurements were carried out in a quartz cell using an electrochemiluminescence detector (MPI-E, Xi’an Remex Analytical Instrument Co., Ltd., Xi’an, China). ECL signal was measured during continuous CV scanning with a potential range from 0 to 1.4 V at a scanning rate of 100 mV/s. The luminescent body was Ru(bpy)_3_^2+^, and the luminescent wavelength is 610 nm [62]. A bias voltage of 400 V was employed. All measurements were performed at room temperature.

### 3.3. Synthesis of AuNPs’ Confined SNF/ITO Electrode

ITO electrodes were pre-treated before use [61]. They were firstly sonicated in a 1 M NaOH aqueous solution to create a negatively charged surface for 10 min, followed by ultrasonic cleaning in acetone, ethanol, and water for 10 min, respectively. Afterward, they were dried at 60 °C before use.

SNF was grown on the pretreated ITO surface using the Stöber-solution growth method using CTAB as the surfactant template [63]. This approach created a dense array of nanochannels that were oriented vertically to the ITO surface. Subsequently, the ITO glasses with SNF on the surface were cut into dimensions of 0.5 cm × 5 cm, and the working electrode area was fixed to 0.5 cm × 1 cm using insulating glue. Given that the nanochannel interiors were filled with surfactant micelle (SM), the resulting electrode was designated as SM@SNF/ITO. SM can be easily removed by immersing the SM@SNF/ITO electrode in HCI (0.1 M in ethanol) for 8 min [63]. The final electrode with an open nanochannel array that was oriented vertically to the surface of the underlying ITO electrode was named as SNF/ITO.

To deposit AuNPs within the nanochannels of SNF, the working electrode was immersed in a 0.5% aqueous solution of HAuCl_4_, and a constant voltage of −0.5 V was applied for 3 s. The AuNPs-modified electrode was obtained after rinsing with ultrapure water.

### 3.4. Fabrication of Aptamosensor

For the covalent immobilization of recognitive aptamer, the outer surface of SNF is firstly derivatized by epoxy groups. To ensure that the epoxyization reaction occurs primarily on the outer surface of SNF rather than inside the nanochannels, an electrode with SM-sealed nanochannels (SM@SNF/ITO) was employed for epoxy derivatization, followed by the removal of SM. Specifically, SM@SNF/ITO was immersed in a 25 mL ethanol solution containing GPTMS (2.26 mM) for 1 h to introduce reactive epoxy groups onto the external surface of the SNF. Then, the obtained electrode was cleaned with ultrapure water followed with removal of SM. The obtained electrode was designated as O-SNF/ITO.

Subsequently, AuNPs were confined within the nanochannels of O-SNF/ITO, through simple electrodeposition [34]. Briefly, O-SNF/ITO was subjected to a 5% HAuCl_4_ solution and electrodeposition was performed at −0.5 V for 3 s, yielding AuNPs@O-SNF/ITO.

For the fabrication of the recognitive interface, AuNPs@O-SNF/ITO was immersed in the CRP aptamer (Apt) solution (0.3 μM in 0.01 M PBS) and incubated at 4 °C for 80 min. This process led to the covalent immobilization of the CRP aptamer, resulting in Apt/AuNPs@O-SNF/ITO. Finally, Apt/AuNPs@O-SNF/ITO was incubated with BSA (10 mg/mL) at 4 °C for 30 min to block non-specific active sites, creating the aptasensor, BSA/Apt/AuNPs@O-SNF/ITO. The fabricated aptasensor was directly stored in a refrigerator at 4 °C when not in use.

### 3.5. ECL Determination of CRP

The ECL intensity before and after CRP binding was assessed by incubating the fabricated aptasensor with different concentrations of CRP at 37 °C for 80 min. Specifically, the aptasensor before (BSA/Apt/AuNPs@O-SNF/ITO) or after CRP binding (CRP/BSA/Apt/AuNPs@O-SNF/ITO) were immersed in a PBS solution (0.01 M, pH = 7.4) containing the ECL emitter (Ru(bpy)_3_^2+^, 10 μM) and co-reactor (TPrA, 3 mM). The ECL signal of the electrode was then recorded. For the analysis of real samples, CRP in human serum (from a healthy male) was determined using a standard addition method. Briefly, human serum was spiked with different concentrations of CRP and then diluted 50-fold with PBS (0.01 M, pH 7.4) before measurement.

## 4. Conclusions

In this work, a simple and label-free ECL aptasensor has been successfully developed for the highly sensitive detection of CRP in biological samples. Large-area SNF-modified electrodes were fabricated on cost-effective and readily available ITO glass, allowing the simultaneous preparation of multiple SNF-modified electrodes. Due to the low cost of both ITO and silica-based SNF, the cost of SNF/ITO is very low (commonly 1 USD/cm^2^). The modified electrode used can be easily miniaturized and patterned. Subsequently, AuNPs were grown in situ within the nanochannels of SNF through electrodeposition. This achievement established a dual ECL signal amplification system based on the efficient enrichment of ECL emitters within SNF nanochannels and the catalytic enhancement by AuNPs. By functionalizing the outer surface of SNF with epoxy groups when the nanochannels was blocked with a surfactant template, a covalently immobilized recognition interface was obtained for aptamer immobilization. In comparison with the traditional sandwich-type immunoassay, e.g., enzyme-linked immunosorbent assay (ELISA), the fabricated aptasensor employs DNA aptamers as recognitive ligands and the immobilization process of aptamers is similar to the immobilization step of antibodies in traditional immunosensors. However, aptamers are cost-effective and exhibit much higher stability than antibodies [64,65]. For CRP detection, the constructed aptasensor exhibited a wide linear range, low detection limit, high selectivity, and excellent storage stability. Furthermore, the aptasensor also demonstrated advantages such as low cost and rapid analysis, resulting in satisfactory recovery rates in CRP detection in serum samples. The aptasensor developed in this study has great promise for potential applications in disposable, portable detection or point-of-care diagnostics.

## Figures and Tables

**Table 1:** Determination of CRP in serum sample using the fabricated aptasensor.

Sample	Added ^a^ (ng/mL)	Found ^b^ (ng/mL)	Recovery (%)	RSD (%, n = 3)
	0.100	0.104	104	1.8
Serum ^c^	1.00	0.947	94.4	1.6
	100	102	102	1.2

^a^ The concentration was obtained after serum dilution. ^b^ The concentration represents the average of measurements on three serum samples. ^c^ The sample was diluted by a factor of 50.

## Data Availability

The data presented in this study are available on request from the corresponding author.

## Supplementary Materials

The following supporting information can be downloaded at: https://www.mdpi.com/article/10.3390/molecules28227664/s1, Table S1: Comparison of CRP detection performance with other sensors.

## References

[1] Boncler M., Wu Y., Watala C. (2019). The multiple faces of C-reactive protein-physiological and pathophysiological implications in cardiovascular disease. Molecules.

[2] Sproston N.R., Ashworth J.J. (2018). Role of C-reactive protein at sites of inflammation and infection. Front. Immunol..

[3] Pepys M.B., Hirschfield G.M. (2003). C-reactive protein: A critical update. J. Clin. Investig..

[4] Yang Y., Xie J., Guo F., Longhini F., Gao Z., Huang Y., Qiu H. (2016). Combination of C-reactive protein, procalcitonin and sepsis-related organ failure score for the diagnosis of sepsis in critical patients. Ann. Intensive Care.

[5] Prajapati A., Verma N., Pandya A. (2020). Highly sensitive vertical flow based point-of-care immunokit for rapid and early detection of human CRP as a cardiovascular risk factor. Biomed. Microdevices.

[6] Macwan I., Aphale A., Bhagvath P., Prasad S., Patra P. (2020). Detection of cardiovascular CRP protein biomarker using a novel nanofibrous substrate. Biosensors.

[7] Rasmussen L.J.H., Schultz M., Gaardsting A., Ladelund S., Garred P., Iversen K., Eugen-Olsen J., Helms M., David K.P., Kjær A. (2017). Inflammatory biomarkers and cancer: CRP and suPAR as markers of incident cancer in patients with serious nonspecific symptoms and signs of cancer. Int. J. Cancer.

[8] Lv Y., Wu R., Feng K., Li J., Mao Q., Yuan H., Shen H., Chai X., Li L.S. (2017). Highly sensitive and accurate detection of C-reactive protein by CdSe/ZnS quantum dot-based fluorescence-linked immunosorbent assay. J. Nanobiotechnol..

[9] Bravin C., Amendola V. (2020). Wide range detection of C-Reactive protein with a homogeneous immunofluorimetric assay based on cooperative fluorescence quenching assisted by gold nanoparticles. Biosens. Bioelectron..

[10] Zhang L., Li H.-Y., Li W., Shen Z.-Y., Wang Y.-D., Ji S.-R., Wu Y. (2018). An ELISA Assay for Quantifying Monomeric C-Reactive Protein in Plasma. Front. Immunol..

[11] Zhong S., Chen L., Shi X., Chen G., Sun D., Zhang L. (2023). Recent advances in electrochemical aptasensors for detecting cardiac biomarkers: A review. Microchem. J..

[12] Tang M.-Q., Xie J., Rao L.-M., Kan Y.-J., Luo P., Qing L.-S. (2022). Advances in aptamer-based sensing assays for C-reactive protein. Anal. Bioanal. Chem..

[13] Sun D., Lu J., Zhang L., Chen Z. (2019). Aptamer-based electrochemical cytosensors for tumor cell detection in cancer diagnosis: A review. Anal. Chim. Acta.

[14] Wang Y., Liu X., Wu L., Ding L., Effah C.Y., Wu Y., Xiong Y., He L. (2022). Construction and bioapplications of aptamer-based dual recognition strategy. Biosens. Bioelectron..

[15] Zhang T., Yang L., Yan F., Wang K. (2023). Vertically-ordered mesoporous silica film based electrochemical aptasensor for highly sensitive detection of alpha-fetoprotein in human serum. Biosensors.

[16] Zhong W., Sczepanski J.T. (2019). A mirror image fluorogenic aptamer sensor for live-cell imaging of microRNAs. ACS Sens..

[17] Valizadeh Shahbazlou S., Vandghanooni S., Dabirmanesh B., Eskandani M., Hasannia S. (2023). Biotinylated aptamer-based SPR biosensor for detection of CA125 antigen. Microchem. J..

[18] Mahyari M., Hooshmand S.E., Sepahvand H., Gholami S., Rezayan A.H., Zarei M.A. (2021). Gold nanoparticles anchored onto covalent poly deep eutectic solvent functionalized graphene: An electrochemical aptasensor for the detection of C-reactive protein. Mater. Chem. Phys..

[19] López L., Hernández N., Reyes Morales J., Cruz J., Flores K., González-Amoretti J., Rivera V., Cunci L. (2021). Measurement of neuropeptide Y using aptamer-modified microelectrodes by electrochemical impedance spectroscopy. Anal. Chem..

[20] Jiang M., Wang M., Lai W., Song X., Li J., Liu D., Wei Z., Hong C. (2023). Construction of electrochemical and electrochemiluminescent dual-mode aptamer sensors based on ferrocene dual-functional signal probes for the sensitive detection of Alternariol. Anal. Chim. Acta.

[21] Liu X., Luo L., Li L., Di Z., Zhang J., You T. (2019). An electrochemiluminescence aptasensor for analysis of bisphenol A based on carbon nanodots composite as co-reaction of Ru(bpy)32+ nanosheets. Electrochim. Acta.

[22] Nikolaou P., Valenti G., Paolucci F. (2021). Nano-structured materials for the electrochemiluminescence signal enhancement. Electrochim. Acta.

[23] Li J., Luo M., Jin C., Zhang P., Yang H., Cai R., Tan W. (2022). Plasmon-enhanced electrochemiluminescence of PTP-decorated Eu MOF-based Pt-tipped Au bimetallic nanorods for the lincomycin assay. ACS Appl. Mater. Interfaces.

[24] Gu W., Wang H., Jiao L., Wu Y., Chen Y., Hu L., Gong J., Du D., Zhu C. (2020). Single-Atom Iron Boosts Electrochemiluminescence. Angew. Chem. Int. Ed..

[25] Xu S., Zhang S., Li Y., Liu J. (2023). Facile synthesis of iron and nitrogen co-doped carbon dot nanozyme as highly efficient peroxidase mimics for visualized detection of metabolites. Molecules.

[26] Chang Q., Huang J., He L., Xi F. (2022). Simple immunosensor for ultrasensitive electrochemical determination of biomarker of the bone metabolism in human serum. Front. Chem..

[27] Zhu Q., Liu H., Zhang J., Wu K., Deng A., Li J. (2017). Ultrasensitive QDs based electrochemiluminescent immunosensor for detecting ractopamine using AuNPs and Au nanoparticles@PDDA-graphene as amplifier. Sens. Actuators B Chem..

[28] Wang L., Liu Y., Yan J., Li H., Tu Y. (2023). Novel electrochemiluminescent immunosensor using dual amplified signals from a CoFe Prussian blue analogue and Au nanoparticle for the detection of Lp-PLA2. ACS Sens..

[29] Gai Q.-Q., Wang D.-M., Huang R.-F., Liang X.-X., Wu H.-L., Tao X.-Y. (2018). Distance-dependent quenching and enhancing of electrochemiluminescence from tris(2,2′-bipyridine) ruthenium (II)/tripropylamine system by gold nanoparticles and its sensing applications. Biosens. Bioelectron..

[30] Zhou Z., Yu F., Ma J. (2022). Nanoconfinement engineering for enchanced adsorption of carbon materials, metal–organic frameworks, mesoporous silica, MXenes and porous organic polymers: A review. Environ. Chem. Lett..

[31] Huang L., Su R., Xi F. (2023). Sensitive detection of noradrenaline in human whole blood based on Au nanoparticles embedded vertically-ordered silica nanochannels modified pre-activated glassy carbon electrodes. Front. Chem..

[32] Zhang C., Zhou X., Yan F., Lin J. (2023). N-doped graphene quantum dots confined within silica nanochannels for enhanced electrochemical detection of doxorubicin. Molecules.

[33] Ding L.H., Su B. (2015). A non-enzymatic hydrogen peroxide sensor based on platinum nanoparticle-polyaniline nanocomposites hosted in mesoporous silica film. J. Electroanal. Chem..

[34] Ding L., Li W., Sun Q., He Y., Su B. (2014). Gold nanoparticles confined in vertically aligned silica nanochannels and their electrocatalytic activity toward ascorbic acid. Chem.-Eur. J..

[35] Liang R., Dong J., Li J., Jin H., Wei M., Bai T., Ren W., Xu Y., He B., Suo Z. (2024). DNAzyme-driven bipedal DNA walker and catalytic hairpin assembly multistage signal amplified electrochemical biosensor based on porous AuNPs@Zr-MOF for detection of Pb^2+^. Food Chem..

[36] Ma X., Qian K., Ejeromedoghene O., Kandawa-Schulz M., Wang Y. (2020). Electrochemical detection of microRNA based on SA-PPy/AuNPs nanocomposite with the signal amplification through catalytic hairpin assembly reaction and the spontaneous catalytic reaction of Fe^3+^/Cu^2+^. Electrochim. Acta.

[37] Zhao J., Duan W., Liu X., Xi F., Wu J. (2023). Microneedle patch integrated with porous silicon confined dual nanozymes for synergistic and hyperthermia-enhanced nanocatalytic ferroptosis treatment of melanoma. Adv. Funct. Mater..

[38] Liu X., Chen Z., Wang T., Jiang X., Qu X., Duan W., Xi F., He Z., Wu J. (2022). Tissue imprinting on 2D nanoflakes-capped silicon nanowires for lipidomic mass spectrometry imaging and cancer diagnosis. ACS Nano.

[39] Cui Y., Duan W., Jin Y., Wo F., Xi F., Wu J. (2020). Ratiometric fluorescent nanohybrid for noninvasive and visual monitoring of sweat glucose. ACS Sens..

[40] Walcarius A. (2021). Electroinduced surfactant self-assembly driven to vertical growth of oriented mesoporous films. Acc. Chem. Res..

[41] Zhou H., Ding Y., Su R., Lu D., Tang H., Xi F. (2022). Silica nanochannel array film supported by ß-cyclodextrin-functionalized graphene modified gold film electrode for sensitive and direct electroanalysis of acetaminophen. Front. Chem..

[42] Zhu X., Xuan L., Gong J., Liu J., Wang X., Xi F., Chen J. (2022). Three-dimensional macroscopic graphene supported vertically-ordered mesoporous silica-nanochannel film for direct and ultrasensitive detection of uric acid in serum. Talanta.

[43] Cui Y., Zhang S., Zhou X., Yan F., Hu W. (2023). Silica nanochannel array on co-electrodeposited graphene-carbon nanotubes 3D composite film for antifouling detection of uric acid in human serum and urine samples. Microchem. J..

[44] Yan F., Su B. (2016). Tailoring molecular permeability of nanochannel-micelle membranes for electrochemical analysis of antioxidants in fruit juices without sample treatment. Anal. Chem..

[45] Lin X., Yang Q., Ding L., Su B. (2015). Ultrathin silica membranes with highly ordered and perpendicular nanochannels for precise and fast molecular separation. ACS Nano.

[46] Zou Y., Zhou X., Xie L., Tang H., Yan F. (2022). Vertically-ordered mesoporous silica films grown on boron nitride-graphene composite modified electrodes for rapid and sensitive detection of carbendazim in real samples. Front. Chem..

[47] Su R., Tang H., Xi F. (2022). Sensitive electrochemical detection of p-nitrophenol by pre-activated glassy carbon electrode integrated with silica nanochannel array film. Front. Chem..

[48] Yang L., Zhang T., Zhou H., Yan F., Liu Y. (2022). Silica nanochannels boosting Ru(bpy)_3_^2+^-mediated electrochemical sensor for the detection of guanine in beer and pharmaceutical samples. Front. Nutr..

[49] Zhang M., Zou Y., Zhou X., Yan F., Ding Z. (2022). Vertically-ordered mesoporous silica films for electrochemical detection of Hg(II) ion in pharmaceuticals and soil samples. Front. Chem..

[50] Zheng W., Su R., Yu G., Liu L., Yan F. (2022). Highly sensitive electrochemical detection of paraquat in environmental water samples using a vertically ordered mesoporous silica film and a nanocarbon composite. Nanomaterials.

[51] Chen H., Huang J., Zhang R., Yan F. (2022). Dual-mode electrochemiluminescence and electrochemical sensor for alpha-fetoprotein detection in human serum based on vertically ordered mesoporous silica films. Front. Chem..

[52] Chen D., Luo X., Xi F. (2023). Probe-integrated electrochemical immunosensor based on electrostatic nanocage array for reagentless and sensitive detection of tumor biomarker. Front. Chem..

[53] Zhou P., Su B. (2022). Enhanced electrochemiluminescence at silica nanochannel membrane studied by scanning electrochemical microscopy. J. Electroanal. Chem..

[54] Villalonga A., Vegas B., Paniagua G., Eguílaz M., Mayol B., Parrado C., Rivas G., Díez P., Villalonga R. (2020). Amperometric aptasensor for carcinoembryonic antigen based on a reduced graphene oxide/gold nanoparticles modified electrode. J. Electroanal. Chem..

[55] Kong D., Zhao J., Tang S., Shen W., Lee H.K. (2021). Logarithmic data processing can be used justifiably in the plotting of a calibration curve. Anal. Chem..

[56] Yang H.J., Kim M.W., Raju C.V., Cho C.H., Park T.J., Park J.P. (2023). Highly sensitive and label-free electrochemical detection of C-reactive protein on a peptide receptor−gold nanoparticle−black phosphorous nanocomposite modified electrode. Biosens. Bioelectron..

[57] Cheng Y.-Y., Feng X.-Z., Zhan T., An Q.-Q., Han G.-C., Chen Z., Kraatz H.-B. (2023). A facile indole probe for ultrasensitive immunosensor fabrication toward C-reactive protein sensing. Talanta.

[58] Rong Z., Chen F., Jilin Y., Yifeng T. (2019). A C-reactive protein immunosensor based on platinum nanowire / titania nanotube composite sensitized electrochemiluminescence. Talanta.

[59] Yang X., Xu Y., Huang X., Hang J., Guo W., Dai Z. (2023). Multicolor iridium(iii) complexes with host–guest recognition motifs for enhanced electrochemiluminescence and modular labeling. Anal. Chem..

[60] Cui C., Lin X., Lv J., Guo H., Shen L., Xiang G., Zhao W., Jiang D. (2023). Electrochemiluminescence resonance energy transfer between Ru(bpy)_3_^2+^@Cu_3_(HHTP)_2_ and GO-Au composites for C-reactive protein detection. Talanta.

[61] Hong D., Kim K., Jo E.-J., Kim M.-G. (2021). Electrochemiluminescence-Incorporated Lateral Flow Immunosensors Using Ru(bpy)_3_^2+^-Labeled Gold Nanoparticles for the Full-Range Detection of Physiological C-Reactive Protein Levels. Anal. Chem..

[62] Yan L., Zhang C., Xi F. (2022). Disposable amperometric label-free immunosensor on chitosan–graphene-modified patterned ITO electrodes for prostate specific antigen. Molecules.

[63] Teng Z., Zheng G., Dou Y., Li W., Mou C.-Y., Zhang X., Asiri A.M., Zhao D. (2012). Highly ordered mesoporous silica films with perpendicular mesochannels by a simple Stöber-solution growth approach. Angew. Chem. Int. Ed..

[64] Hassan E.M., DeRosa M.C. (2020). Recent advances in cancer early detection and diagnosis: Role of nucleic acid based aptasensors. TrAC Trend Anal. Chem..

[65] Negahdary M. (2020). Aptamers in nanostructure-based electrochemical biosensors for cardiac biomarkers and cancer biomarkers: A review. Biosens. Bioelectron..

