# Supplementary material for: Electrochemiluminescence Aptasensor with Dual Signal Amplification by Silica Nanochannel-Based Confinement Effect on Nanocatalyst and Efficient Emitter Enrichment for Highly Sensitive Detection of C-Reactive Protein
Source: Molecules. 2023 Nov 19;28(22):7664. doi: 10.3390/molecules28227664 (PMC10675231; doi:10.3390/molecules28227664)
Supplement: Supplementary file 1 [file molecules-28-07664-s001.zip › molecules-2649504-supplementary.pdf]

Table S1 Comparison of CRP detection performance with other sensors.

**Electrode/Material**	**Method**	**Recognitive ligand**	**Linear range (ng/mL)**	**LOD (pg/mL)**	**Ref.**
Ab/TiNTs/PtNWs/ITO	ECL	Antibody	0.05-6.25	11	[58]
GO-AuNPs-Ab_2/CRP/BSA/Ab_1/ Ru@$Cu_3(HHTP)_2$/GCE	ECL	Antibody	0.005-50	0.26	[60]
$Ru(bpy)_3^{2+}$@AuNPs-Ab_2/CRP/Ab_1/SPE	ECL	Antibody	0.01-1000	4.6	[61]
Ir(III)-β-CD/Ab_2/CRP/Ab_1-MB/	ECL	Antibody	$1\text{-}1\times10^4$	72	[59]
Apt/AuNPs/GO/PDES/GCE	EIS	Aptamer	0.001-50	0.3	[18]
GBP3/AuNPs@BP@PDA/Au	SWV	Peptide receptor	0-36	700	[56]
BSA/Ab/indole/AuNPs/SPCE	DPV	Antibody	$0.1\text{-}1\times10^5$	30	[57]
BSA/Apt/AuNPs@O-SNF/ITO	ECL	Aptamer	0.01-1000	7.4	This work

TiNTs: titania nanotubes; PtNWs: platinum nanowire; GO: graphene oxide; Ab_2: the second CRP antibody; Ab_1: the first CRP antibody; Ru@$Cu_3(HHTP)_2$: $Ru(bpy)_3^{2+}$-loaded Cu_3(hexahydroxytriphenylene); GCE: glassy carbon electrode; AuNPs: gold nanoparticles; SPE: screen-printed electrode; Ir(III)-β-CD: Ir(III) compound and β-cyclodextrin complex; MB: magnetic bead; Apt: CRP aptamer; PDES: poly deep eutectic solvents; GBP: gold binding peptide; BP: black phosphorus; PDA: tethered polydopamine; SWV: square wave voltammetry; SPCE: screen-printed carbon electrode.; DPV: differential pulse voltammetry.
